# Cultivation of microalgae–bacteria consortium by waste gas–waste water to achieve CO_2_ fixation, wastewater purification and bioproducts production

**DOI:** 10.1186/s13068-023-02409-w

**Published:** 2024-02-15

**Authors:** Wenwen Kong, Jia Kong, Shuo Feng, TianTian Yang, Lianfei Xu, Boxiong Shen, Yonghong Bi, Honghong Lyu

**Affiliations:** 1 Tianjin Key Laboratory of Clean Energy and Pollution Control, School of Energy and Environmental Engineering, Hebei University of Technology, Tianjin, 300401 People’s Republic of China; 2 Hebei Engineering Research Center of Pollution Control in Power System, Hebei University of Technology, Tianjin, 300401 People’s Republic of China; 3 State Key Laboratory of Freshwater Ecology and Biotechnology, Institute of Hydrobiology, Chinese Academy of Sciences, Wuhan, 430072 People’s Republic of China

**Keywords:** Microalgae–bacteria, CO_2_ fixation, Waste gas, Wastewater, Mechanism, Products of microalgae

## Abstract

The cultivation of microalgae and microalgae–bacteria consortia provide a potential efficient strategy to fix CO_2_ from waste gas, treat wastewater and produce value-added products subsequently. This paper reviews recent developments in CO_2_ fixation and wastewater treatment by single microalgae, mixed microalgae and microalgae–bacteria consortia, as well as compares and summarizes the differences in utilizing different microorganisms from different aspects. Compared to monoculture of microalgae, a mixed microalgae and microalgae–bacteria consortium may mitigate environmental risk, obtain high biomass, and improve the efficiency of nutrient removal. The applied microalgae include *Chlorella* sp., *Scenedesmus* sp., *Pediastrum* sp., and *Phormidium* sp. among others, and most strains belong to Chlorophyta and Cyanophyta. The bacteria in microalgae–bacteria consortia are mainly from activated sludge and specific sewage sources. Bioengineer in CBB cycle in microalgae cells provide effective strategy to achieve improvement of CO_2_ fixation or a high yield of high-value products. The mechanisms of CO_2_ fixation and nutrient removal by different microbial systems are also explored and concluded, the importance of microalgae in the technology is proven. After cultivation, microalgae biomass can be harvested through physical, chemical, biological and magnetic separation methods and used to produce high-value by-products, such as biofuel, feed, food, biochar, fertilizer, and pharmaceutical bio-compounds. Although this technology has brought many benefits, some challenging obstacles and limitation remain for industrialization and commercializing.

**Graphical Abstract:**

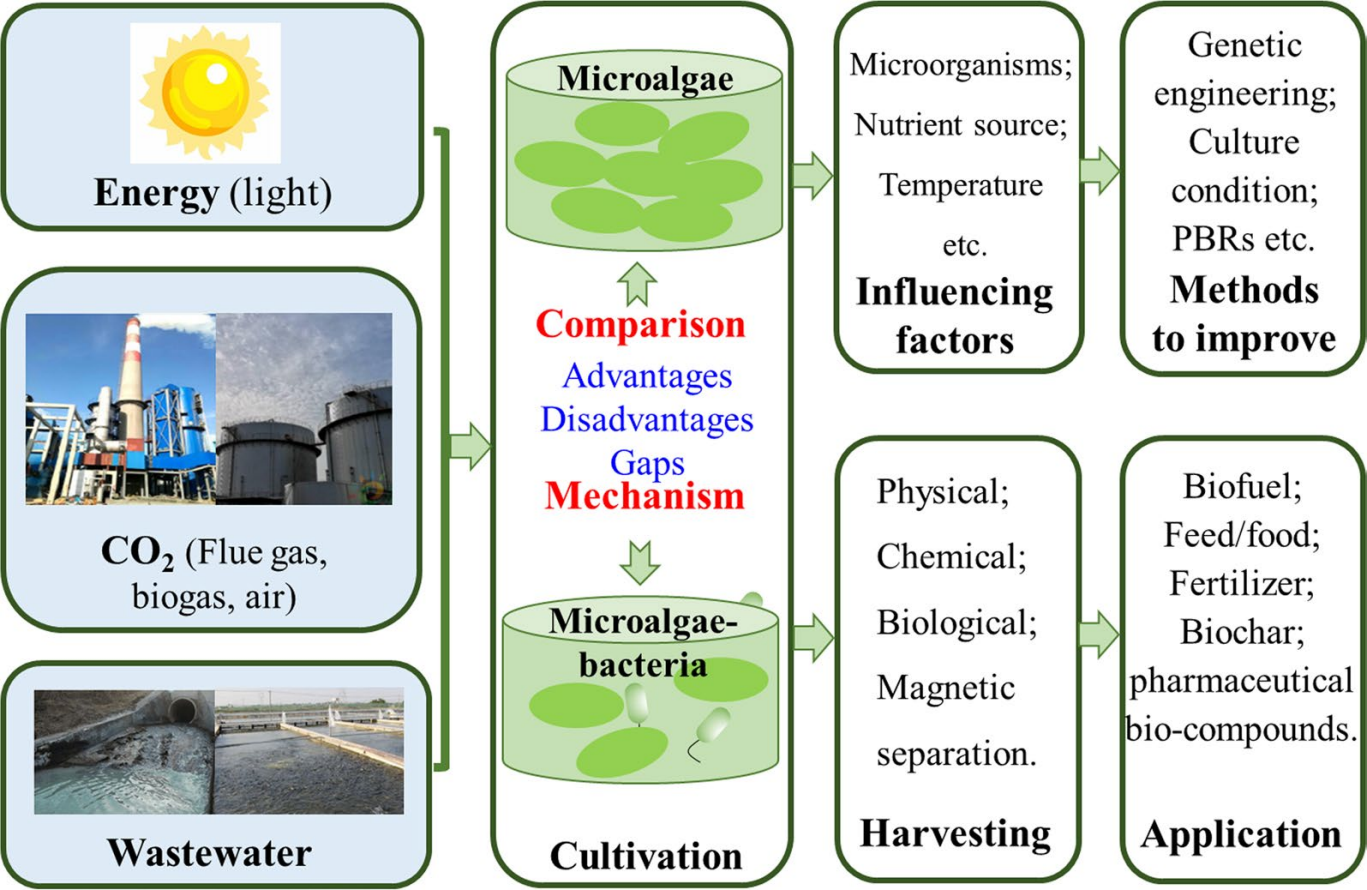

## Introduction

**Fig. 1:**
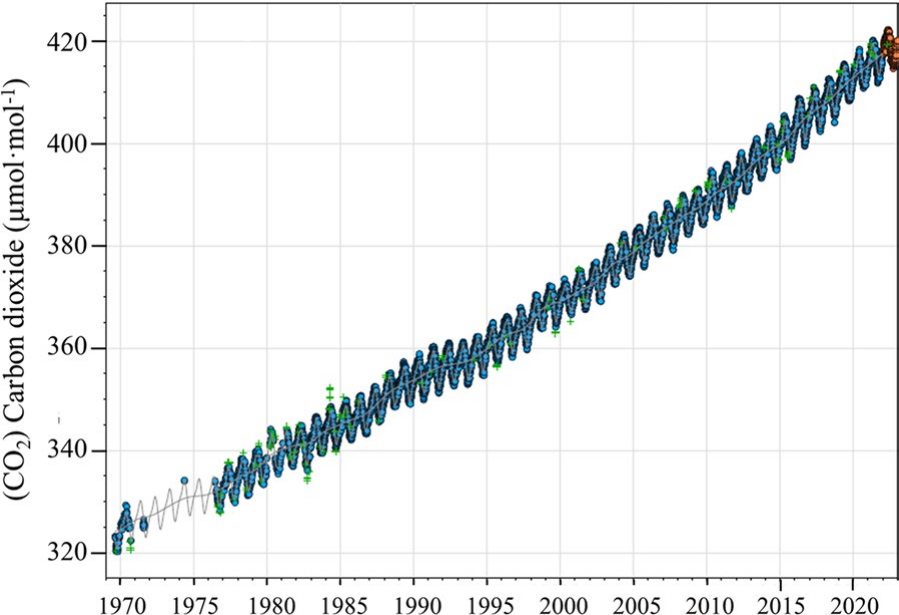
CO_2_ concentrations in in Mauna Loa, Hawaii, United States [2]

A large amount of carbon dioxide (CO_2_) has been emitted into the atmosphere, which exacerbates global warming and the greenhouse effect [1]. CO_2_ concentration in the atmosphere has reached to 420.0 ppm in 2022 in Mauna Loa, Hawaii, United States (as shown in Fig. 1) [2]. In this background, global carbon reduction and neutrality have become worldwide topics [3]. Microalgae are the main microorganisms for photosynthesis on Earth, and their carbon (C) consumption accounts for nearly 50% of global CO_2_ fixation. The application of microalgae to fix CO_2_ is considered an efficient strategy to eliminate the atmospheric CO_2_ concentration [4], and has great potential in combating global warming due to its green economy as well as pollution-free nature [5, 6].

Unreasonable disposal of wastewater discharges a large amount of nitrogen (N), phosphorus (P), carbon (C), heavy metals and other pollutants into freshwater bodies, causing the disturbance of aquatic ecosystems and destruction of species diversity. Considering the high cost of traditional microalgal cultures (i.e., using culture medium and water), replacing the culture medium with wastewater showed great promise while in effective removal of pollutants from wastewater [7, 8]. Thus, to cut down the cost of microalgae cultivation, waste gas–wastewater has been used to cultivate microalgae as nutrient source. For example, with suitable culture conditions, cultured microalgae could fix 450 tons of CO_2_, 25 tons of N, and 2.5 tons of P per hectare per year while simultaneously producing 200 tons of microalgal biomass [9].

Microalgae are grown as monocultures in many studies, and applications using specific algal strains desired for harvest could result in a high yield of high-value products [10]. For instance, swine wastewater and waste CO_2_ were applied in cultivating *Chlorella vulgaris* in an integrated semi-continuous system, when wastewater renewal rate was 80%, the highest productivity of *Chlorella vulgaris* was obtained at 3% CO_2_ [11]. Then, *Chlorella vulgaris* biomass can be utilized for lipid extraction [12, 13]. However, due to the microalgae culture system is easily contaminated by undesired microorganisms in the industrialization or outdoor cultivation process, the culture system is difficult to maintain using a single species. Thus, consortia composed of mixed microalgae or microalgae–bacteria were proposed. Compared with a monoculture of microalgae, mixed cultures of microalgae or microalgae–bacteria are potential alternatives for tackling various pollutants due to the more robust biological system, and could improve the performance of wastewater purification [14, 15]. To strengthen the resistance of microalgae, improve the treatment effect of wastewater, and enhance the CO_2_ fixation ability, the novel microalgae–bacteria partnership system and its regulatory mechanism need to be explored [16].

There are huge variety of natural species of microalgae and bacteria on Earth [17]. Comparing the applications of microalgae and microalgae–bacteria grown in wastewater and waste gas is meaningful and important for the selection of suitable microorganisms, improvement of their CO_2_ fixation ability, and their industrialization. Moreover, it has been reported that microalgae and bacteria compete for survival in limited nutrients and space, thereby influencing their application scope and efficiency [16, 18].

In the above background, this paper reviews and compares the applications of single microalgae, mixed microalgae, and microalgae–bacteria consortia in fixing CO_2_ and treating wastewater, and emphasizes the gaps between the existing studies and industrialization. The influencing factors for cultivating these microorganisms along with methods to improve the performance are further discussed. It also focuses on revealing their mechanisms of CO_2_ fixation and nutrients removal. Moreover, the harvest method of microalgae and the high-value products from microalgae were concluded. Finally, this paper provides advice for future works to cultivate microalgae–bacteria consortium by waste gas–waste water for CO_2_ fixation, wastewater purification and bioproducts production.

## Microalgae and microalgae–bacteria consortia

### Monoculture of microalgae

Chlorophyta (green algae) are the major microalgae that have been extensively studied, including *Chlorella* sp*.*, *Chlorococcum* sp., *Pseudokirchneriella subcapitata*, *Scenedesmus* sp*.*, *Coelastrum* sp. and *Nannochloropsis gaditana* (Table 1)*.* When cultivating *Chlorella vulgaris* in simulated municipal wastewater injected with CO_2_, the maximal CO_2_ fixation rates (56.26–85.72 mg CO_2_·L^−1^·d^−1^) and nutrient removal rates (96.12–99.61%) at 10% CO_2_ were obviously higher than those at air condition [19]. The effects of 6–16% CO_2_ on the CO_2_ fixation rate and nutrient removal rate by *Coelastrum sp.* were also studied, and the maximal CO_2_ fixation rate (302 mg·L^−1^·h^−1^), total nitrogen (TN) removal rate (84.01%) and total phosphorus (TP) removal rate (100%) were obtained at 12% CO_2_ [20]. TN and TP removal rates were 97.80% and 95.60% caused by *Scenedesmus obliquus*, respectively, and the maximal CO_2_ fixation rate was 26.45 mg·L^−1^·h^−1^ [21]. N and P could also be uptaken by *Pseudokirchneriella subcapitata* with removal rates of 100% and 51.30%, respectively, and the CO_2_ fixation rate reached 264 mg·L^−1^·d^−1^ [22].

**Table 1:** Cultivation of microalgae by waste gas–waste water to fix CO_2_ and remove nutrients

	Wastewater	CO_2_ (% *v/v*)	*R*_*CO2*_	*R*_*N*_	*R*_*P*_	References
Chlorophyta						
*Chlorella* sp.; *Chlorococcum* sp.	Industrial wastewater	1–10	187.65 94.68	100^b^	98.8 85.8^c^	[7]
*Chlorella vulgaris*	Municipal wastewater	0.04–20	318	99 ^a^	87.95	[8]
*Chlorella sp. UKM2*	Palm oil mill effluent	10–25	120	80.9	–	[25]
*Chlorella vulgaris* *Scenedesmus obliquus*	Municipal wastewater	0.038–5	140.91 123.82	93.4 91.5	94.1 91.3	[26]
*Chlorella sp.* L166	Soybean processing wastewater	0–10	28.6%	96.07	95.55	[27]
*Chlorella kessleri*	Synthetic wastewater	0–10	83.88	99	88	[28]
*Chlorella* sp*. GD*	Aquaculture wastewater	0.038–10	2333	90	99	[29]
*Chlorella vulgaris MBFJNU-1*	Swine slurry	1–20	454	74	87	[30]
*Chlorella vulgaris* *Chlorella pyrenoidosa* *Scenedesmus obliquus* *Scenedesmus dimorphu*	Effluent from wastewater treatment plant	10	120 250 270 200	92.13–97.38	> 80.43	[31]
*Chlorella vulgaris*	Steel mill wastewater	10.9–11.3	13.52	77^a^	61^c^	[32]
*Chlorella vulgaris* *Pseudokirchneriella subcapitata*	Simulated domestic effluent	0.038	471 264	99.0 100	67.6 51.3	[22]
*Chlorella vulgaris*	Simulated municipal wastewater	10	170.98–220.92	> 97.64	> 97.64	[12]
*Scenedesmus obliquus*	Secondary effluent	0.03–15	26.45 ± 1.51	97.8	95.6	[33]
*Scenedesmus obliquus U169*	Tequila vinasses	0.038–25	910	75.96	-	[34]
*Tetradesmus obliquus*	Secondary effluent	5–15	106.6–275.1	77.57–91.47	> 98	[35]
Cyanophyta						
*Spirulina platensis*	Municipal wastewater	2.5–15	378	–	94.0	[18]
*Microcystis aeruginosa* *Synechocystis salina*	Simulated domestic effluent	0.038	384 384	100 100	37.9 41.1	[22]

*R*_*CO2*_ (mg CO_2_·L^−1^·d^−1^), CO_2_ fixation rate; *R*_*N*_ (%), removal rate of nitrogen (N);* R*_*P*_ (%), removal rate of phosphorus (P); a, ammonia nitrogen; b, nitrate and nitrite; c, orthophosphate

*Spirulina platensis*, *Microcystis aeruginosa* and *Synechocystis salina*, belonging to Cyanophyta (blue algae), have also been effectively cultivated in waste gas–waste water for CO_2_ fixation as well as wastewater purification (Table 1). Almomani et al. reported that the CO_2_ fixation rate of *Spirulina platensis* ranged from 62 to 378 mg CO_2_·L^−1^·d^−1^ at 2.5–15% CO_2_, with a maximum at 10% CO_2_. The NO_3_–N removal efficiencies from secondary effluent were 57.6–58% by *Spirulina platensis* [18]. In the study of Gonçalves et al., *Microcystis aeruginosa* and *Synechocystis salina* showed the potential to fix CO_2_ (400 mg CO_2_·L^−1^·d^−1^) and remove N (12.53–19.63 mg N·L^−1^·d^−1^), but the P removal rates were low (1.16–1.62 mg P·L^−1^·d^−1^, 37.9–41.1%) [22].

In addition, when *Phormidium valderianum* BDU 20041 was grown in ossein effluent injected with 15% *v/v* CO_2_, it fixed 56.4 mg CO_2_·L^−1^·d^−1^ and removed 66.35% N and 35.66% P [23]. In pharmaceutical wastewater with 0.038% CO_2_, the CO_2_ fixation rate, removal rate of NO_3_–N, and removal rate of PO_4_^3−^–P by *Tetraselmis Indica* BDU 123 reached 89 mg CO_2_·L^−1^·d^−1^, 81.6% and 94.87%, respectively [24].

### Mixed microalgae cultivation

Mixed microalgae culture is a technique in which two or more species of high-yield microalgae are cultivated in a culture system to obtain biomass. The coculture of multiple microalgae improved the biomass and CO_2_ fixation rate through the interaction or synergistic effects among microalgae [18, 36]. Table 2 shows that Chlorophyta are important components of mixed microalgae. For instance, *Chlorella vulgaris*, *Botryococcus braunii* and *Spirulina platensis* were cocultured in treated sewage and 1% *v/v* CO_2_ [37]. The maximum of CO_2_ fixation rate, N removal rate, P removal rate and biomass productivity of the cocultured system reached 22,400 mg CO_2_·m^−3^·d^−1^, 91%, 100% and 48,300 mg·d^−1^·m^−2^, respectively [37]. Microalgal consortiums, including *Chlorella* sp., *Scenedesmus* sp., *Sphaerocystis* sp., and *Spirulina* sp., isolated from a wastewater treatment plant with 50% CO_2_, also showed high CO_2_ sequestration efficiency (53–100%) and high nutrient removal efficiency [38].

**Table 2:** Cultivation of mixed microalgae by waste gas–waste water to fix CO_2_ and remove nutrients

Microalgae	Wastewater	CO_2_ (% *v/v*)	*R*_*CO2*_	*R*_*N*_	*R*_*P*_	*P*_*B*_	*N*	References
Mixed algal culture from different wastewater after treatment process	Primary effluent	2.5–15	492 mgC·L^−1^·d^−1^	58.1	–	0.246–0.384 g_dw_·L^−1^·d^−1^	287 mg·L^−1^	[18]
	Secondary effluent		362 mgC L^−1^ d^−1^	95.7	–		200 mg·L^−1^	
	Septic tank effluent		470 mgC L^−1^ d^−1^	99.6	–		230 mg·L^−1^	
Mixed consortia of fresh water and storm water algae	Nature fresh water	Air and coal fired flue gas (0.038, 1, 3, 5.5 CO_2_%)	–	–	–	–	37.73–59.75 mg·L^−1^·d^−1^	[41]
*Scenedesmus obliquus*	Artificial wastewater	0.038–10	–	80^a^	–	–	30–210 10^5^ cells·mL^−1^	[42]
*Scenedesmus* sp. LX1 and *Chlorella ellipsoidea* YJ1; *Haematococcus* and *Chlorella ellipsoidea* YJ1; *Scenedesmus* sp. LX1 and* Haematococcus*	Secondary effluent	0.038	–	–	–	–	183.0 mg·L^−1^	[39]
							204.0 mg·L^−1^	
							277.0 mg·L^−1^	
*Chlorella vulgaris* TISTR-8580 *Botryococcus braunii* NIES-2199 *Spirulina platensis*	Simulated treated sewage	1	22,400 mg·m^3^·day^−1^	91.0	100.0	48.3 g·(d·m^2^)^−1^	923.0 mg·L^−1^	[37]
*Chlorella* sp., *Scenedesmus* sp. *Sphaerocystis* sp., *Spirulina* sp.	Domestic wastewater	2–100	291.0 mg·g^−1^	39.0	59.0	0.114 g·(L·d)^−1^	850.0 mg·L^−1^	[38]
Mixed microalgae in wastewater plant: cyanobacteria, diatoms, *Scenedesmus* sp., *Chlorella* sp.	Untreated urban wastewater	20	24.6 mg L^−1^·min^−1^	100.0	100.0	28.3 g·(d·m^2^)^−1^	–	[36]

*R*_*CO2*_, CO_2_ fixation rate; *R*_*N*_ (%), removal rate of nitrogen; *R*_*P*_ (%), removal rate of phosphorus; *P*_*B*_, biomass productivity; *N*, microalgal biomass; DIC, dissolved inorganic carbon

Mixed microalgae cultivation showed better performance on biomass production, CO_2_ fixation, wastewater treatment than microalgal monocultivation. The growth characteristics of *Scenedesmus* LX1, *Chlorella ellipsoidea*, and *Hematococcus pluvialis* in monoculture and pairwise mixed culture in urban secondary effluent were investigated, it was found that the biomass and specific growth rate of mixed culture of pairwise algal species were higher than their single species [39]. Local mixed microalgae (including yellow‒green, green, blue‒green algae, etc.) from different wastewaters after the treatment process (primary effluent, secondary effluent, and septic tank effluent) were used to fix CO_2_ and purify wastewater [18]. The maximal biomass productivity and CO_2_ fixation rate of mixed microalgae in the study of Almomani et al. were 0.384 g_dw_·L^−1^·d^−1^ and 0.460 g C·L^−1^·d^−1^, respectively, which were obviously higher than those of *Spirulina platensis* [18]. In a study by Johnson et al., a polyculture of algal species (*Chlorella*, *Scenedesmus*, *Chlorococcus*, and *Phaeodactylum tricornutum*) was more stable than the cultures of single microalgal species, less susceptible to the external environment, and could reduce the risks of microalgal biomass harvesting and wastewater remediation [40]. A raceway pond (200 L), operating outdoors, was designed and used to cultivate mixed microalgae such as *Scenedesmus* sp. and *Chlorella* sp. in untreated urban wastewater injected with 20% CO_2_ [36]. CO_2_ gas was supplied continuously at different flow rates of 0.2–5.0 L·min^−1^ during the daytime [36]. The maximum CO_2_ removal rate (24.6 mg·L^−1^·min^−1^) and microalgae biomass productivity (28.3 g·d^−1^·m^−2^) were reached when the gas flow rate was 1.0 L·min^−1^ [36].

### Microalgae–bacteria consortium cultivation

From Table 3, the bacteria in the microalgae–bacteria consortium involved in not only activated sludge but also functional microorganisms from specific sewage sources, and Chlorophyta are the most commonly used microalgae in the consortium. When *Spongiochloris* was cultivated with bacteria in local petroleum wastewater-injected air (0.038% CO_2_), the microalgal-specific growth rate, biomass productivity, COD removal rate, petroleum hydrocarbon removal rate and maximal CO_2_ bio-fixation rate reached 0.87 d^−1^, 1.5 g·L^−1^·d^−1^, 97%, 99% and 2921 mg·L^−1^·d^−1^, respectively, as the cultivation progressed [43]. A microalgae–bacteria consortium could grow under simulated flue gas from a power plant and achieved effective removal of CO_2_ (4.7–18.4 mg CO_2_·L^−1^·d^−1^), SO_x_ (99%) and NO_x_ (87%) [44]. *Scenedesmus* was inoculated into sterilized wastewater and unsterilized wastewater-injected with 10% CO_2_ for cultivation, and it was found that the COD removal rate in unsterilized wastewater group was 90%, much higher than that of sterilized wastewater (42%) [45]. This result implies that microalgae in the consortium are responsible for fixing CO_2_, while bacteria generally utilize organic carbon.

**Table 3:** Cultivation of microalgae–bacteria consortium by waste gas–waste water to fix CO_2_ and remove nutrients

Microorganisms	Wastewater and gas	*R*_*CO2*_	*R*_*N*_	*R*_*P*_	*R*_*COD*_	Biomass	*R*_*pol*_	References
*Tetradesmus obliquus* PF3 and bacteria in sewage	0.038% CO_2_ 10% CO_2_	551 mg CO_2_·L^−1^·d^−1^	93 ± 3 81 ± 1	99 95	90 ± 3 65 ± 2	1.8 g·L^−1^ 0.9 g·L^−1^	–	[45]
*Spongiochloris* sp. and *Hydrocabonoclastic*	Petroleum wastewater 0.038% CO_2_	2921 mg·L^−1^·d^−1^	–	–	97	1.5 ± 0.3 g·L^−1^·d^−1^	–	[43]
Microalgae (four strains) and aerobic activated sludge	Primary treated sewage Flue gas (12% CO_2_, 289 ppmv NO, 197 ppmv SO_2_)	4.7–18.4 mg CO_2_·L^−1^·d^−1^	–	–	–	0.153–0.181 g·L^−1^·d^−1^	87% for NO_x;_ 99% for SO_2_	[44]
*Chlorella vulgaris* (FACHB-8) and Endophytic bacteria S395-1 and S395-2	Biogas slurry Biogas (62.17 ± 2.44% CH_4_, 34.21 ± 1.29% CO_2_, 0.54 ± 0.03% O_2_, 3.07 ± 0.21% H_2_O)	68.13% ± 1.69%	88.31 ± 4.19	88.21 ± 4.51	88.29 ± 5.03	50–250 μg·L^−1^	–	[46]
Microalgae and algae and bacteria (including *Scenedesmus* spp., *Cyanobacteria*) isolated from wastewater	Wastewater Air and CO_2_ (99%)	–	~ 99	~ 99	–	94.3 ± 7.9 mg·L^−1^·d^−1^	–	[52]
*Chlorella pyrenoidosa and* Native bacterial microbial	Municipal wastewater and landfill leachate treatment	65.8 mg·L^−1^·d^−1^	–	–	–	1.58 g·L^−1^	–	[53]
*Chlorella PY-ZU1 and bacteria* (*from anaerobic digestion effluent*)	Undiluted anaerobic digestion effluent of swine manure 15% *v/v* CO_2_	–	73%^a^	95%	79%	4.81 g·L^−1^ 601.2 mg·L^−1^·d^−1^	35.7–90.0% for Heavy metals	[54]
*Chlorella vulgaris* *Chlorella vulgaris* and* Ganoderma lucidum* *Chlorella vulgaris* and* Activated sludge*	Biogas slurry Biogas (25.27%, 35.08%, 45.36%, 55.17% CO_2_)	68.37 79.11 79.06	69.12 85.69 84.17	66.36 86.17 83.79	68.71 86.08 84.28	0.174 0.431 0.429 g·L^−1^·d^−1^	–	[55]
*Chlorella* sp. and *Cupriavidus necator*	Culture medium (with phenol); 1% CO_2_	–	–	–	–	0.45–0.50 g/L	100% for phenol	[56]
*Spirulina platensis* and H_2_S-oxidizing bacterial consortium and Activated sludge	Mineral salt medium; 30% CO_2_, 69.5–70% N_2_, 0–5000 ppm_v_	95%	–	–	–	1.2 g·L^−1^	100% for H_2_S	[57]
*Chlorella* sp. and aerobic sludge	Mineral salt medium; Biogas	285 mg CO_2_·L^−1^·d^−1^	–	–	–	–	> 98% for H_2_S	[48]
Tree bark consortium; Eukaryotic; *Scenedesmus quadricauda*	Aquaculture effluent: Biogas digestate = 90: 10	52.958%	87.227	100^b^	12.292^c^	1.46 g/L	–	[58]
		40.497%	61.346	73.154^b^	18.455^c^	0.86 g/L	–	
		39.097%	78.693	75.391^b^	− 14.889^c^	0.86 g/L	–	
Tree bark consortium; Eukaryotic; *Scenedesmus quadricauda*	Aquaculture effluent: Biogas digestate = 75: 25	90.714%	56.627	21.543^b^	− 3.473^c^	0.74 g/L	–	
		85.809%	83.205	54.126^b^	− 8.980^c^	0.29 g/L	–	
		89.504%	52.037	1.393^b^	− 11.366^c^	0.19 g/L	–	
Tree bark consortium; Lake water consortium; Pre-adapted tree bark consortium	Aquaculture effluent: Biogas digestate = 95: 5	55.125%	91.287	100^b^	− 6.076^c^	1.10 g/L	–	
		46.293%	87.626	100^b^	− 52.733^c^	1.05 g/L	–	
		55.760%	90.574	100^b^	− 26.791^c^	1.15 g/L	–	
Tree bark consortium; Lake water consortium; Pre-adapted tree bark consortium	Aquaculture effluent: Biogas digestate = 90: 10	80.446%	77.987	100^b^	25.207^c^	1.27 g/L	–	
		69.592%	72.122	100^b^	2.766^c^	1.02 g/L	–	
		83.588%	66.114	100^b^	− 6.334^c^	1.30 g/L		
Tree bark consortium; Lake water consortium; Pre-adapted tree bark consortium	Aquaculture effluent: Biogas digestate = 80: 20	95.775%	33.928	40.401^b^	− 1.340^c^	0.86 g/L		
		92.653%	39.702	75.353^b^	− 4.227^c^	0.99 g/L		
		98.466%	27.637	68.861^b^	− 20.032^c^	1.16 g/L		
*Picochlorum* sp. and *Halospirulina* sp. and Sulphur oxidizing bacteria	Mineral salt medium Biogas (30% *v/v* CO_2_, 0.5% *v/v* H_2_S)	44.5–50.0%	N–NO_3_^−^, 52 ~ 55	P–PO_4_^3−^, 12 ~ 29	–	23–129 mg·L^−1^·d^−1^	99.5% for H_2_S	[59]
*Chlorella vulgaris–Ganoderma lucidum*–endophytic bacteria (*S395-2*) *Scenedusmus obliquus–Pleurotus ostreatus–*endophytic bacteria (*S395-2*)	Biogas slurry Biogas	56.29–64.87% 54.79–62.37%	68.37–79.15; 67.08–75.36	74.58–83.65; 70.31–80.43	62.27–76.89; 60.27–72.57	0.085–0.163 g·L^−1^·d-^1^ 0.079–0.149 g·L^−1^·d^−1^	–	[49]

*R*_*CO2*_, CO_2_ fixation rate; *R*_*N*_ (%), removal rate of nitrogen; *R*_*P*_ (%), removal rate of phosphorus; *R*_*COD*_ (%), removal rate of organic carbon; *P*_*B*_, microalgal biomass productivity; *R*_*pol*_, removal rate of other pollutants in wastewater or gas; a, ammonia nitrogen (NH_4_^+^–N); b, orthophosphate (PO_4_^3−^P); c, dissolved organic carbon (DOC);dw, dry weight

Moreover, the cultivation process of microalgae–bacteria was the only technology capable of simultaneously upgrading biogas by removing CO_2_ and H_2_S while recovering nutrients from digestates (in Table 3). The endophytic bacteria S395-1 and S395-2 (different genera) were co-cultivated with *Chlorella vulgaris*, and the consortium had removal efficiencies of 88.29%, 88.31%, 88.21%, and 68.13% for COD, N, P, and CO_2_, respectively [46]. Alcantara et al. [47] and Lebrero et al. [48] reported that the CO_2_ removal by a microalgae–bacteria consortium in pond or bubble column photobioreactors was 55–62%. The performance of biogas slurry purification by *Chlorella vulgaris*–*Ganoderma lucidum*–endophytic bacteria (S395-2) symbionts was better than that of biogas slurry purification by *Scenedesmus obliquus*–*Pleurotus ostreatus*-S395-2 symbionts [49].

The decrease in CO_2_ content of biogas (accounting for 25–50% of biogas by volume) will lead to a decrease in transportation costs and an increase in biogas energy content. Although the use of algal–bacterial consortia has achieved promising results, the low CO_2_ mass transfer rate of this technology limits biogas bioconversion to biomethane [50]. Using natural light as photosynthetic active radiation daily (~ 433 μE·m^−2^·s^−1^), cultivating microalgae–bacteria consortium in high-rate ponds can efficiently remove COD within 10 days and remove nutrients within 26 days, without extra cost for CO_2_ addition [51].

### Gaps in applications

#### Gaps in monoculture of microalgae

Microalgal cultures are grown as monocultures in many studies. An important reason is that the application of microalgal monocultures is easy to conduct in the laboratory and reveal the feasibility of a scheme. Another primary reason is that specific algal strains desired for harvest could obtain a high yield of high-value products. However, it is difficult for microalgae to maintain a pure culture state under natural conditions, and much time and energy are needed for purification and preservation of the microalgae, such as in the disinfection or sterilization of sewage. Moreover, the system stability is poor, and the microalgae are easily killed by foreign species pollution, requiring high culture equipment, which is not conducive to practical application.

#### Gaps in mixed microalgae and microalgae–bacteria consortium cultivation

Since diversity would improve biomass stability, the ability of large-scale culture systems of mixed microalgae and microalgae–bacteria consortia to resist the mutation of environmental conditions (temperature, illumination, etc.) was higher than that of single microalgae. The use of high-yield microalgal species with different optimal conditions could expand the control range of the culture conditions, thereby reducing the maintenance cost of the culture system.

However, there are several important controversial issues in the applications of mixed microalgae and microalgae–bacteria cultivation. First, it is difficult to obtain a high yield of specific high-value bioproducts, and the microalgal biomass is easily affected by the microalgal species [18]. Second, studies on microalgal biotechnology research investigate already known species. Among the huge variety of natural species—thousands—there is still a wide scope for selecting fast-growing naturally occurring species at specific geographic locations and profiting from their metabolic capabilities. In addition, because of the complexity of the microorganisms in the consortium, the stability is difficult to control, and the pathways of nutrient removal and CO_2_ fixation in the microalgal–bacterial consortium are easily changed under at different conditions. Ultimately, the efficiency of wastewater treatment and CO_2_ fixation is influenced [60].

## Effective factors

The efficiencies of wastewater treatment and CO_2_ fixation by microalgae or microalgal–bacterial consortia are easily changed under different conditions. The effective factors can be classified as microorganisms and cultivation conditions in general.

### Microorganisms

Microalgae strains with rapid growth rates and dense populations were picked up for production and capturing CO_2_ from flue gas. As shown in Tables 1–3, microalgae used to fix CO_2_ from waste gas and purify wastewater are *Chlorella* sp., *Scenedesmus* sp., *Pediastrum* sp., *Phormidium* sp. etc., and most of strains belong to Chlorophyta and Cyanophyta. The CO_2_ fixation rates of Chlorophyta and Cyanophyta are higher than those of others, such as *Phormidium valderianum* BDU 20041.

The bacteria in microalgae–bacteria consortia are mainly from activated sludge, digestion effluent and other specific sewage sources. However, COD concentrations increased when some microalgae–bacteria consortia were utilized inappropriately [58]. Thus, the CO_2_ fixation and nutrients removal efficiency should be optimized by identifying suitable strains of microalgae and bacteria to be co-culture. Studies on which bacteria in microalgal–bacterial consortia can efficiently fix carbon under different conditions should be conducted systematically.

### Nutrient sources

#### Waste gas

To provide sufficient carbon source for microalgae growth, air and treated waste gas are bubbled as CO_2_ sources into the algal body of water. As shown in Tables 1–2, the CO_2_ concentrations from the atmosphere or flue gas used to cultivate microalgae are in range of 0.038–25%. From Table 3, biogas also can be implemented in microalgae–bacteria consortium cultivation, and the applied CO_2_ concentrations were in the range of 0.038–99%. To adapt high concentrations of CO_2_ (20%), *Chlamydomonas* increased the cell concentrations, *Nostoc* increased cell size, and *Chlorella* increased both concentrations and size of cells [61]. When microalgae–bacteria consortium was cultivated, the simulated flue gas with 12% CO_2_ was injected into primary treated sewage at the gas flow rate of 0.025 *vvm* [44]. The gas flow rate was low, because CO_2_ fixed by the consortium was not only from external gas but also from bacterial decomposition [44]. Thus, adequate flow rate and concentrations of CO_2_ are important to cultivate adequate microorganisms.

However, the presence of NO_x_, SO_x_ and heavy metals (such as Hg) in flue gas may have a negative effect on microalgae growth. For example, the biomass of *Chlorella vulgaris* grown in wastewater decreased with the increasing of Hg concentrations (10–30 μg·Nm^−3^) in flue gas [12]. The CO_2_ fixation rate and growth of *Chlorella* sp. were improved when the added amount of NO_x_ was appropriate, but they were decreased when the added amount of NO_x_ was excessive [62]. Due to the pH of culture was easily affected by the acidic gases (such as NO_x_ and SO_2_), Cheng et al. [5] reported that controlling volume flowrate of flue gas is crucial, and the effect of these acidic gases’ components can be neglected if an optimized volume flowrate is applied.

#### Wastewater

Multiple types of wastewaters, such as municipal wastewater, industrial wastewater, palm oil mill effluent, aquaculture wastewater, steel mill wastewater, ossein effluent, and pharmaceutical wastewater, have been utilized to cultivate single species of microalgae (in Table 1). For mixed microalgae, they have rarely been grown in industrial wastewater or pharmaceutical wastewater (in Table 2). From Tables 1 and 2, the composition of wastewater affects microalgal growth as well as the efficiency of wastewater purification due to the utilization of nutrients and other compounds by microalgae. For different wastewater, the removal rates of N and P can reach 58.1–100% and 37.9–100%, respectively (Tables 1 and 2).

Both common wastewater and biogas slurry have been used to cultivate microalgae–bacteria consortia. According to Table 3, both N and P could be removed with high efficiency (60–100%) in most cultivation systems, but the nutrients removal efficiencies are low when the consortia were grown in biogas digestate. The removal rates of N in 80% aquaculture effluent and 20% biogas digestate by the Tree bark consortium, Lake water consortium, and preadapted tree bark consortium were 27.64–39.70% [58]. The removal rate of P in 80% aquaculture effluent and 20% biogas digestate by the Tree bark consortium, Eukaryotic consortium, and *Scenedesmus quadricauda* reached 1.39–54.13% [58]. Moreover, the removal efficiencies of COD by microalgae–bacteria consortium were negative [58].

In wastewater, N deficiency directs the carbon flux generated during photosynthesis towards the production of fatty acids, but the cell division is low in this technology which ultimately leads to a decrease in productivity of biomass and fatty acids [63]. When N is too high, it can have toxic effects on the microalgae. Some of the effects caused by P deficiency are similar to those obtained in N-deficient cultures, influencing the cellular content of metabolite production.

## Methods to improve the performance

### Optimization of microorganisms

Microalgae and microalgae–bacteria consortia composed of more microalgal and bacterial species have been explored, such as the selection of native microalgae and bacteria [18, 44, 45, 52]. The selected native microorganisms can adapt to environment more easily. In general, complex ecosystem containing microalgae, algae, and bacteria from waste source (such as walls of the secondary clarifier, etc.) were collected, and they were passed through a laboratory paper filter to remove filamentous bacteria and zooplankton from reactor [18, 44, 45, 52]. Then, the filtered solution was inoculated into a suitable medium with suitable environment, and the main microalgae or bacterial genus present in the medium were screened after cultivation [18, 44, 45, 52].

**Fig. 2:**
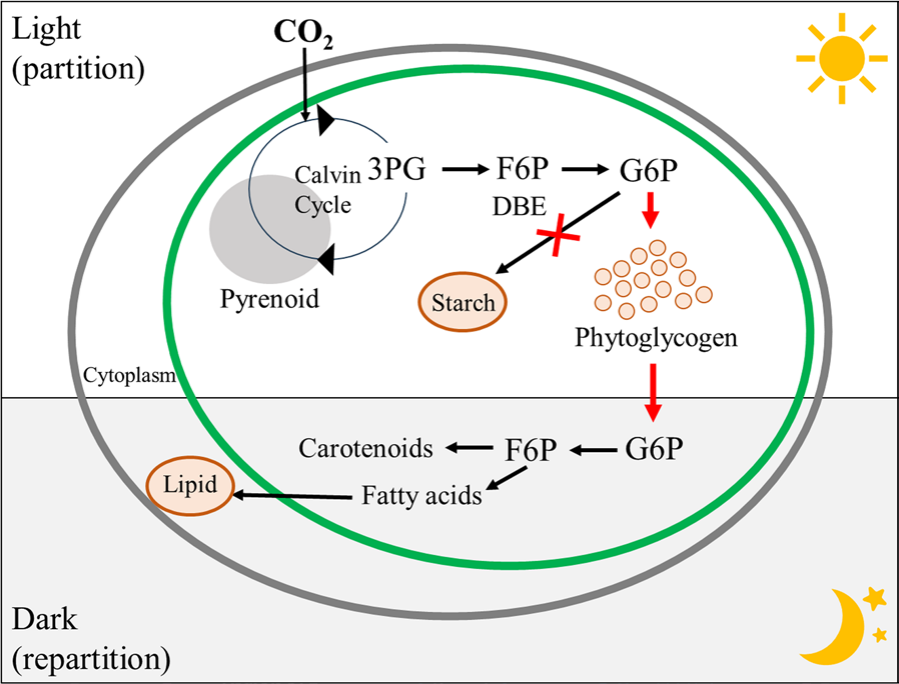
Carbon partition/repartition model for starch debranching enzyme (DBE)-deficient microalgae under light/dark conditions [10]

Moreover, to achieve improvement of CO_2_ fixation or a high yield of high-value products, bioengineer and mutation in CBB cycle in microalgae cells have been studied [46]. For example, when a Rubisco activase was induced in *Nannochloropsis oceanica*, the over expression of Rubisco was elevated, the biomass growth rate and lipid productivity was increased by 32% and 41%, respectively [64]. In Yang et al.’s study, the aldolase gene from *Synechocystis* sp. PCC 6803 (sFBA) was cloned and fused with cTP sequence to be targeted into the chloroplast of *C. vulgaris* [65]. The overexpression of gene encoding aldolase in *Chlorella vulgaris* cells can significantly enhance the efficiency of CO_2_ fixation [65]. In Kato et al.’s study, in the ISA gene encoding an isoamylase-type starch debranching enzyme of Chlamydomonas sp. KOR1, a 2.0 kb sequence covering the initiation codon through part of the N-terminal early set domain is deleted and substituted by a 0.6 kb sequence [10]. The CO_2_ fixation rate by starch debranching enzyme-deficient microalgae was improved through the above process, and its CO_2_ fixation mechanism and carbon partition/repartition model are shown in Fig. 2 [10]. CO_2_ is fixed through the CBB cycle under light conditions, and CO_2_ are mainly captured in the form of water-soluble phytoglycogen (Fig. 2). When microalgae were grown in dark, the phytoglycogen were degraded and converted into intermedia metabolites, which in turn serve as substrate for the synthesis of lipid and carotenoid [10].

### Optimization of cultivation conditions

Microalgal growth and biochemical composition can be influenced by temperature. For example, increasing temperature can decrease the content of total lipid in microalgae cells while improve the content of neutral lipid [63, 66]. Depending on strain, region and season, microalgae can typically grow in-between 15 and 40 ℃ [63], and the suitable temperature for algal cultivation was in range of 21–30 ℃. However, in industrial application, when microalgae were cultivated in industrial flue gases, the temperature of flue gases can reach up to 70 ℃ [67]. Thus, to prevent the inhibition effect caused by high temperature, the temperature should be decreased through cooling or high temperature dominant microalgal strains should be selected.

Illumination (including illumination time and illumination intensity) is another crucial parameter influencing microalgal growth [63, 68]. In microalgae cells, photons can be absorbed and converted to chemical bound energy instantaneously [63, 68]. Long illumination time is beneficial to cultivating microalgae, but only suitable light intensity is beneficial to reaching up highest biomass productivity and CO_2_ fixation rate. In Zhang et al.’s study, the NH_4_^+^–N removal efficiency is higher for 24 h compared to 6 h illumination time with same other culture conditions [69]. When light intensity beyond optimum level, it resulted in photo inhibition, reduced biomass productivity, CO_2_ fixation rate as well as the PUFA content of algae [63, 70].

In fact, in addition to temperature and illumination, many other operation parameters should be optimized, such as N concentrations, P concentrations, organic carbon concentrations, to alleviate the limitations or inhibitory effects of unsuitable cultivation conditions on microalgae growth [14]. *Tetradesmus obliquus* was cultured in municipal wastewater supplemented with 0–100 mg·L^−1^ NH_4_Cl, the maximum biomass and maximal CO_2_ fixation rates were obtained at 100 mg·L^−1^ NH_4_Cl [35]. The supplement of organic carbon also promotes microalgal growth and helps in diverting the carbon flow towards the accumulation of lipid or starch [63]. Due to many factors can affect microalgae growth, microalgae culture conditions were optimized through artificial intelligence [71]. For instance, Yew et al. compared the effects of waste molasses and commercial BG-11 medium on microalgae cultivation by the artificial intelligence algorithm, and determined the optimal culture condition [72].

### Addition of phytohormone

Phytohormone have been applied in resistance of microalgal cells to stress such as SO_2_, NO_x_ and heavy metals from complicated waste gas or wastewater. For example, in Wang et al.’s study, to resist the adversity from NO in coal fired flue gas and improve CO_2_ fixation efficiency by *Chlorella* sp., 500 μM spermidine was supplied into microalgal culture system [73]. The result showed that *Chlorella* sp. biomass productivity was increased by 30.5% under 327 ppm NO [73]. Similarly, when *Chlorella vulgaris* was cultured in 10% CO_2_ gas with 30 μg·m^−3^ Hg, indole-3-acetic acid can alleviate the toxicity of Hg on *Chlorella vulgaris*, ultimately resulting in enhanced chlorophyll synthesis rates and biomass [12]. Zhao et al. also reported that phytohormones aided *Tetraselmis cordiformis* to enhance their growth under high ammonia stress [74]. In sum, phytohormones can be used to cultivate microalgae under complicated waste water and waste gas.

## Mechanism of microalgae and microalgae–bacteria consortia application

### Relationships among microorganisms

Cells in microalgal consortia interact with each other through allelopathy, growth resource competition, and cell contact, thereby presenting three relationships: promotion, neutrality, and inhibition [75–77]. Two possible reasons may explain why mixed culture can promote the biomass production of microalgae. On one hand, microalgae in mixed culture can release allelochemicals that aggregated nutrient ions and promote biomass accumulation. On the other hand, the demand of different microalgae for nutrients forms a complementary relationship, and mixed algal species can improve the utilization of nutrient resources.

**Fig. 3:**
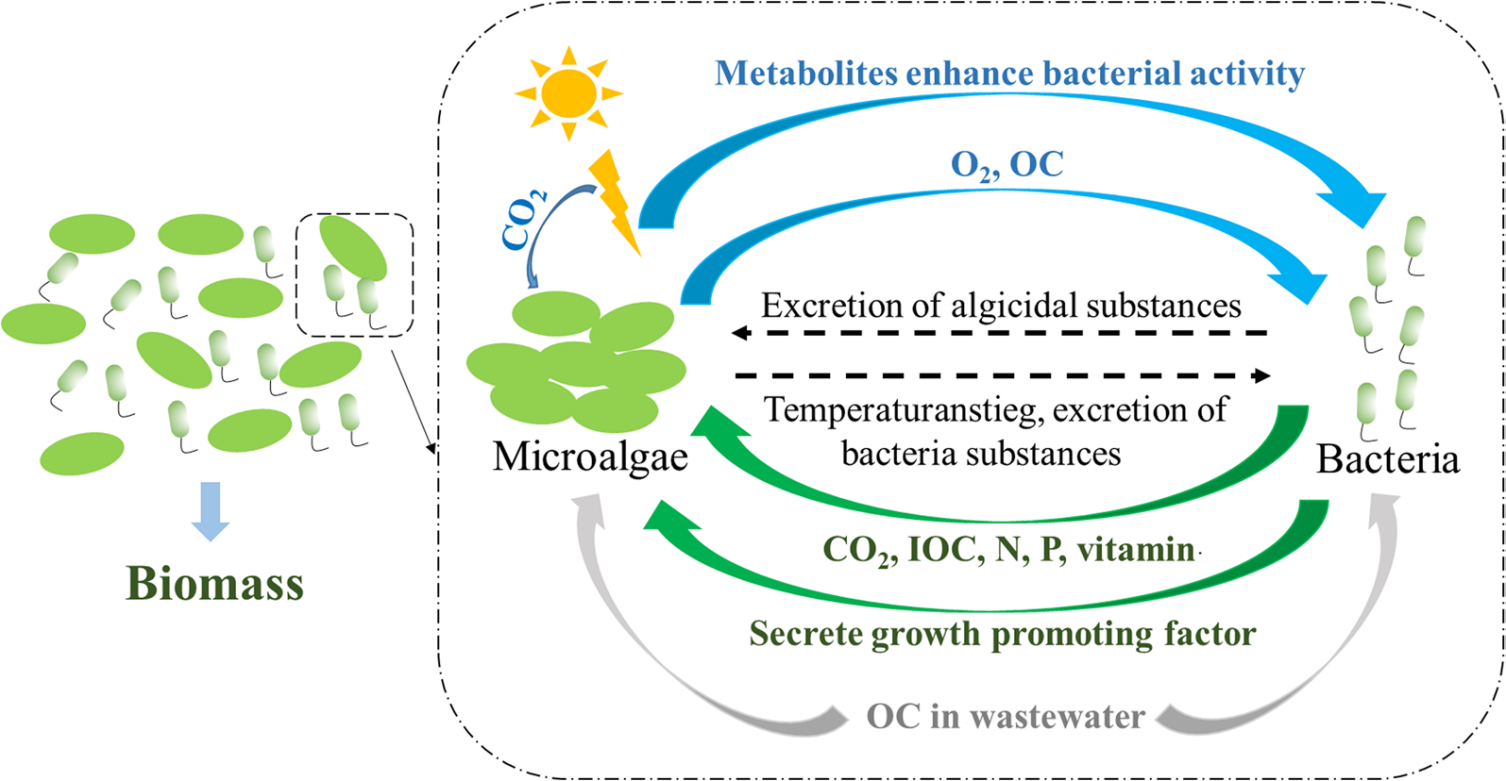
Interaction in microalgal–bacterial consortia (OC, organic carbon; IOC, inorganic carbon; N, nitrogen; P, phosphorus)

The interaction between microalgae and bacteria is complex and mainly includes an improvement relationship and an inhibitory relationship. The inhibitory relationship between microalgae and bacteria is caused by their competition for nutrients and toxins released to inhibit their activities [78]. The improvement relationship is the main relationship when CO_2_ is fixed by microalgae–bacteria consortia, and is mainly manifested in several aspects. Microalgae produce O_2_ through photosynthesis (Fig. 3), increasing the dissolved oxygen content in the water, which is more conducive to the growth of aerobic bacteria [79]. Meanwhile, bacteria oxidize and decompose organic matter for respiration, and promote microalgal growth by creating a favorable microenvironment and providing CO_2_, nutrients, vitamins, phytohormones or volatile organic compounds [80–83]. Moreover, microalgae could serve as a habitat for bacteria, protect them from adverse environmental conditions, and release extracellular polymeric substances to promote bacterial growth [84]. The long-term application of microalgae–bacteria consortium may result in gene transfer to promote their growth [83].

To improve the ability of CO_2_ fixation with nutrient removal by mixed microalgae or microalgae–bacteria consortia, a promotion relationship among microorganisms is necessary. However, the relationship among cells is impacted by their species. Selection of appropriate microalgae and bacteria for mixed culture can effectively improve the biomass yield and CO_2_ fixation rate. At the same time, due to the inherent complexity of the water environment, the influence of other organisms cannot be excluded when explaining the interactions, resulting in conflicting results from some experiments.

### ***Mechanism of CO***_***2***_*** fixation***

**Fig. 4:**
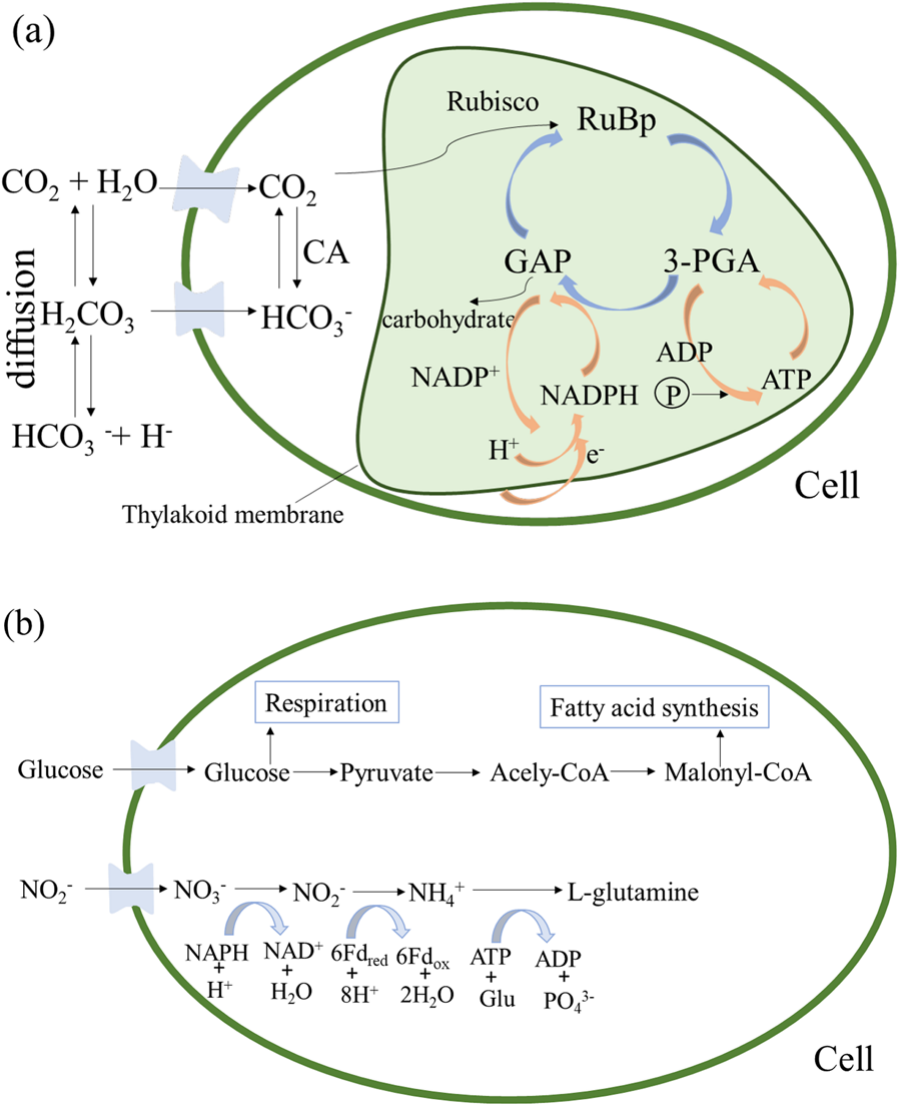
CO_2_ and phosphorus fixation mechanism of microalgae (**a**); Mechanism of organic carbon and nitrogen absorbed by microalgae (**b**)

For most species of microalgae, 1,5-diphosphate ribulose carboxylase/oxygenase (Rubisco) enzymes that catalyze CO_2_ fixation have low affinity for CO_2_ [85], and they only use CO_2_ as substrate. These microalgae can actively absorb HCO_3_^−^ and convert it into CO_2_ under the catalysis of carbonic anhydrase (CA enzyme) for Rubisco fixation (in Fig. 4a) [85]. To increase the CO_2_ concentration in cells and adapt to the change in inorganic carbon concentration, the cells would form a CO_2_ concentration mechanism (CCM), which can increase the CO_2_ concentration of the carboxylase site to 1000 times that of the surrounding environment and form a local high concentration of CO_2_ [86].

The Calvin–Benson–Bassham (CBB) cycle (i.e., Calvin cycle) is the best-known pathway for CO_2_ assimilation in microalgae cells [87–89]. In Fig. 4, CO_2_ in wastewater enters the cell through an inorganic carbon pump and is transported from the cytoplasm to chlorophyll. Then, under the catalysis of Rubisco, CO_2_ is combined with pentose sugar to form 3-phosphoglyceride (3-PGA) to achieve carbon fixation during the CBB cycle. In has been found that, during CO_2_ fixation process, the activity of key enzymes (e.g., Rubisco) or transcription of the *cbb* gene are expected to be improved [90].

In terms of microalgae–bacteria consortia, the mechanisms of CO_2_ fixation are also CCM and CBB and mainly occurred in microalgal cells (Fig. 4a). The progress of CO_2_ fixation is regulated when microalgae are co-cultivated with bacteria [56]. In the study by Yi et al. when *Chlorella* sp. was cultivated with immobilized *Cupriavidus necator*, the expression of most genes related to light reactions and encoding antenna proteins were upregulated to varying degrees [56]. Moreover, most enzymes involved in the C3 pathways were also upregulated in *Chlorella* sp. in the consortium, and it indicates that the fixed CO_2_ amounts were increased [56].

### Mechanism of organic carbon removal

Microalgae are able to directly utilize organic carbon (such as glucose, ethanol, and glycerol) in wastewater as a carbon source through heterotrophy [91]. As illustrated in Fig. 4b, after glucose (as organic carbon) is transported into the cell through the sugar transporter on the algal cell membrane, it can undergo phosphorylation reaction with adenosine triphosphate (ATP) under the catalysis of hexokinase or glucokinase to generate glucose-6-phosphate and ADP [91]. Then, glucose-6-phosphate and ADP enter the glycolysis metabolic pathway, and generate the final product pyruvate. After that, pyruvate is oxidized into CO_2_ and H_2_O through the tricarboxylic acid cycle reaction and electron transport chain and generates ATP. Pyruvate flows through acetyl-CoA into the fatty acid elongation reaction. Glycerol enters algal cells through free diffusion, is phosphorylated by ATP to form 3-phosphoglycerate, forms pyruvate through glycolysis, and then enters the TCA cycle [92].

When a microalgae–bacteria consortium was utilized to treat wastewater, the system was generally in mixotrophic mode. As shown in Fig. 3, the bacteria in the consortium degrades the pollutants in wastewater, and the metabolites produced by bacteria during this process would promote microalgae growth. At the same time, microalgae secretions including carbohydrates, proteins, and fats served as main carbon sources for bacteria growth. The photosynthesis of microalgae produces O_2_, enhances the content of dissolved oxygen in the wastewater, thereby promoting the uptake of organic matter by bacteria and reducing the COD in the wastewater [83]. Microalgae in consortia could also assimilate organic carbon, as described in Figs. 3 and 4a [93]. However, it was noted that this process would decrease the potential for microalgal CO_2_ fixation. In other words, when microalgae were used to fix CO_2_, the utilization of organic carbon in wastewater is limited. With the development of detection technologies, such as high-throughput and missing isotopes analyses, it will be possible to build a mature microalgal–bacterial consortium with a cleaner interaction mechanism and more controllable effects.

### Mechanism of N removal

N plays a vital role in microalgae photosynthesis, participating in the synthesis of organic N, such as amino acids, chlorophyll, energy transfer molecules (ATP and ADP) and genetic components (DNA and RNA). It has been found that the mechanism of N removal is mainly assimilation by microalgae during the cultivation of either microalgae or microalgae–bacteria consortia [15, 93, 94]. As ammonia oxidizing bacteria, *Nitrosomonadaceae* in the microalgae–bacteria consortium are responsible for the nitrification process, but the contribution of bacteria in the process of bioremediation of wastewater is only in range of 1–3% [93]. In a study by Choi et al., microalgae were added into media containing nitrifying bacteria, the results showed that the nitrification rate was reduced despite the near complete removal of NH_4_^+^–N from the system, which also indicates algae were responsible for the removal of NH_4_^+^–N [95].

N in wastewater exists in the form of NO_3_^−^, NH_4_^+^, urea, etc. The utilization pathway of NH_4_^+^ is shorter than other forms of N (such as NO_3_^−^, NO_2_^−^) and requires less energy, and it is preferentially assimilated by microalgae [96]. Any forms of inorganic nitrogen have to be transported into the cells to consume. The consumption of any inorganic nitrogen source requires it to be transport into the cells, which is mediated by an energy-dependent-specific permease in each case. Microalgae convert inorganic N into NH_4_^+^ for utilization through an assimilation process, which is then reduced into two steps by enzymes (Fig. 4b). First, nicotinamide adenine dinucleotide phosphate (NADH) formed by photoreaction is employed as an electron donor to catalyze the transfer of two electrons from NO_3_^−^ to NO_2_^−^, and then NO_2_^−^ is reduced to NH_4_^+^ by nitrite reductase and ferredoxin. Finally, glutamate (Glu), reduced NH_4_^+^ and ATP are combined to generate glutamine under the catalysis of glutamine synthase.

**Fig. 5:**
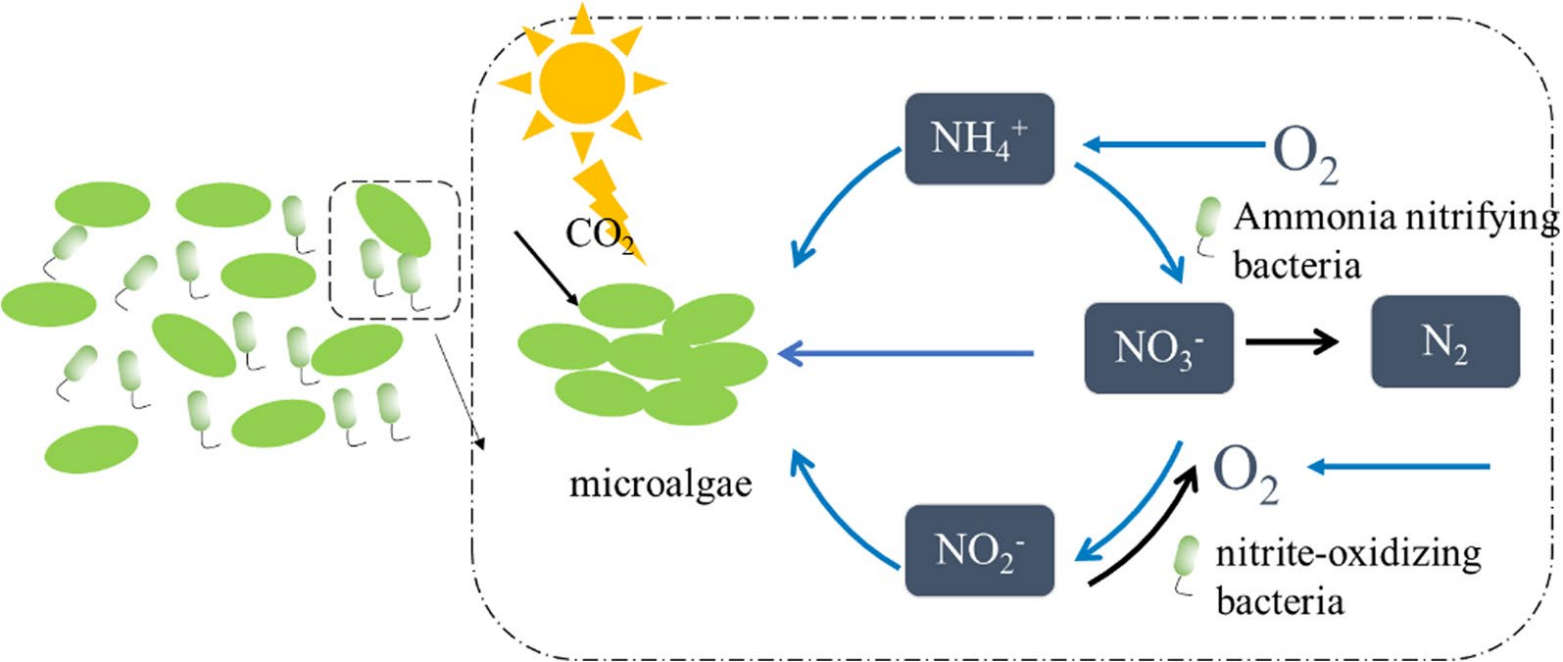
Nitrification and denitrification process (The blue line is nitrification and the black line is denitrification)

The removal of N by the microalgae–bacteria also related to oxidative degradation by bacteria (Fig. 5). As shown in Fig. 5, nitrification is accomplished by adding oxygen from microalgae or gas into water, thereby converting ammonia to nitrate. Under the action of nitrate reductase, NO_3_^−^–N in sewage is reduced to NO_2_^−^–N, and then further reduced to NH_4_^+^–N. NH_4_^+^–N is further utilized by microalgae. In contrast, denitrification occurs in an anoxic environment. Some facultative aerobic heterotrophs in the consortium such as *Bacillus* are added to reduce nitrate and nitrite to nitrogen [80]. Moreover, some metabolites, as enzyme activators, were secreted by co-cultivated bacteria, and a synergistic mechanism between microalgae and bacteria in the enzymology was found [60]. In a study by Wang et al., N-related enzymatic activities in the photosynthesis pathway of *Chlorella* were detected [60]. The results showed that the activities of nitrate reductase (NR), nitrite reductase (NiR), glutamine synthetase (GS), and glutamate synthetase (GOGAT) were improved by 94.2%, 57.5%, 58.6%, and 79.4% caused by the addition of *Exiguobacterium*, respectively [60].

### Mechanism of P removal

The process of P removal from wastewater by microalgae is mainly divided into assimilation and chemical precipitation. The assimilation process means that the P absorbed by cells is converted into organic compounds such as nucleic acids, phospholipids and ATP through multiple phosphorylation pathways, such as oxidation, phosphorylation, photosynthesis etc. [97]. In the process, microalgae often preferentially absorb the inorganic ions H_2_PO_4_^−^ and HPO_4_^2−^ [97, 98]. As reported, a large amount of assimilated P is applied in the production of ATP from ADP, accompanied by a form of energy input, as indicated in Fig. 4a [94]. In the chloroplast, Pi participates in organic binding during photophosphorylation, as ATPases release proton gradients into the substrate; in the stroma, ATP is consumed through the CBB cycle. Consequently, a sufficient amount of P may be one of the parameters for obtaining higher CO_2_ fixation rates.

Chemical precipitation is affected by pH and dissolved oxygen in wastewater. P precipitation may occur when the oxygen concentration is high or the pH exceeds 8.0. When algae perform photosynthesis, CO_2_ is consumed, which increases the wastewater pH. Consequently, in wastewater, the volatilization of NH_3_ and NH_4_^+^ increases, phosphate and calcium ions form calcium phosphate precipitates under high pH conditions, thereby achieving the effective removal of N and P [99]. However, when CO_2_ gas is injected into wastewater, the pH of wastewater remains relatively low (pH < 6.5), and the effects of pH on the removal rate can be ignored [19].

In terms of microalgae–bacteria consortia in aerobic environments, it is found that *Deviosa* sp. and *Bdellovibrio* sp. are the phosphate accumulation bacteria [100]. However, after the wastewater treatment, *Deviosa* sp. accounts for less than 1% of all microorganisms [93, 101]. Due to the percentage of phosphate accumulating bacteria in microorganisms is small, it could be concluded that most phosphate were removed by the microalgae [93, 102]. Microalgae and bacteria can secret polysaccharides, phosphate as well as phosphate hydrolyzed from organophosphorus can also be adsorbed on the surface by forming hydrogen bonds with extracellular polysaccharides. At the same time, similar to the inorganic form, organophosphorus could be combined with functional groups of extracellular polymers, adsorbed to microalgal–bacterial consortium, and then further transformed.

## Harvest and application of microalgal biomass

### Harvest of microalgal biomass

**Fig. 6:**
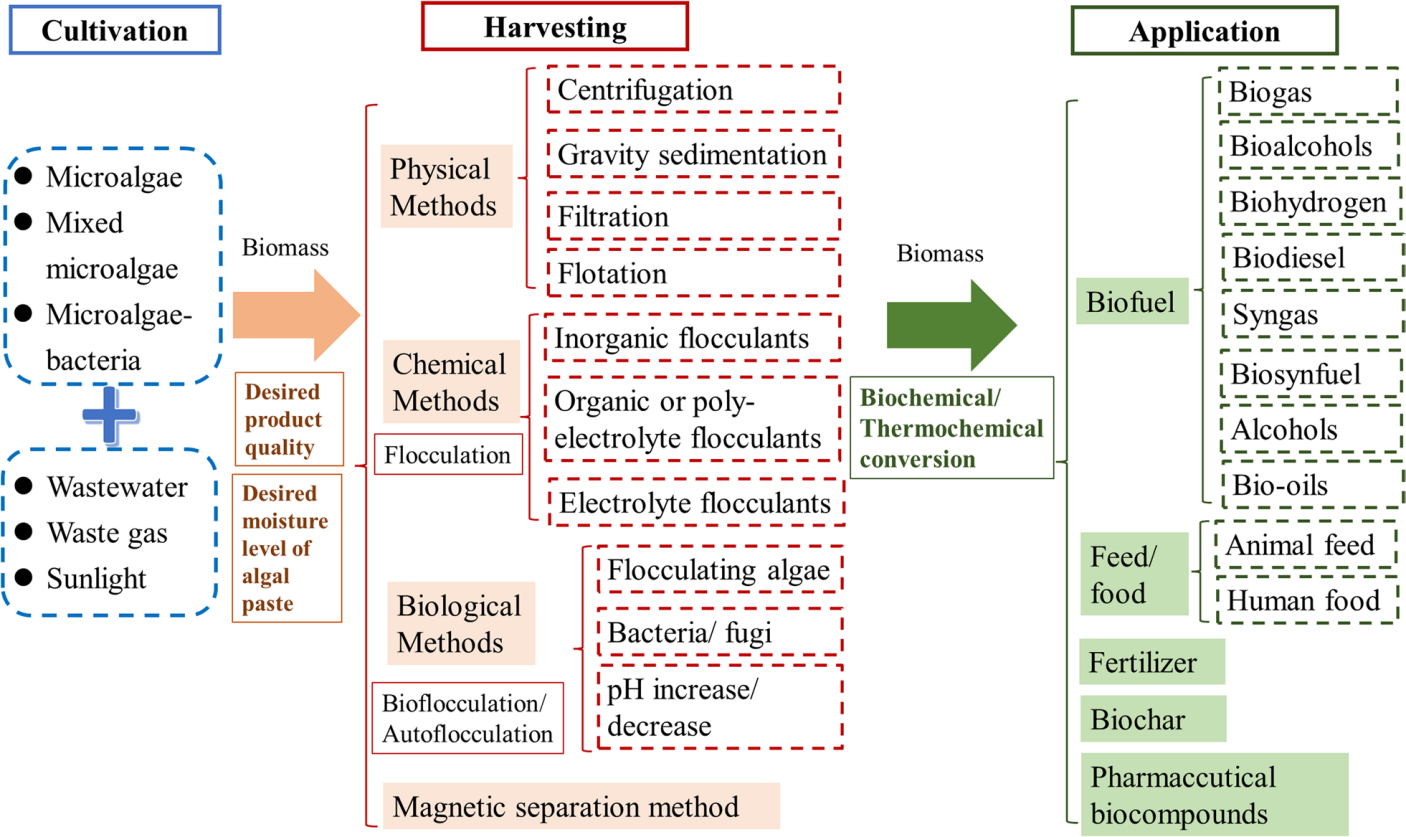
Harvesting and application of microalgae biomass

Microalgal cells are often in a relatively stable suspended state in a culture system, and the sedimentation rate is low. Thus, microalgae cells are difficult to achieve separation through gravity sedimentation and easily clogging the reactor. The harvesting cost of microalgal cells is high, accounting for 20–30% of their biomass production cost [103, 104]. Currently, the methods of microalgae collection are mainly divided into two types: batch collection (flocculation, flotation/gravity setting) and thickening (centrifugation, filtration) (as depicted in Fig. 6) [105–107]. The collection method should be selected according to the desired moisture level of microalgal paste and product quality [103, 108]. For instance, sedimentation/flocculation was used for producing low-value products from microalgae, while centrifugation is suitable for producing high-value products [103]. The percentage of dry matter content in microalgae paste can reach up to 25% through centrifugation [108].

However, most of these techniques have disadvantages of high operating cost, secondary pollution, and low long-term operating efficiency [109, 110]. For instance, centrifugation and filtration are effective methods for collecting microalgae cells, but the costs are very high. In contrast, bioflocculation followed by gravity sedimentation or screening, is a rapid, simple and cost-effective method for harvesting microalgal biomass in large-scale [111].

Biological flocculation is a process in which microalgal cells flocculate with the assistance of microorganisms or their metabolites [13, 106, 112]. During this period, microorganisms aggregate to form large flocs, which are settled by gravity without the addition of any chemical flocculants [113]. Thus, the use of microalgae–bacteria consortia can increase the sedimentation rate of microalgae in culture system to a rate much higher than that of single microalgae. The flocs that bacteria attach to the surface of microalgal cells play an important role in flocculation, enhancing the floc volume of the microalgal cells, so that the flocs are large enough to settle [33]. At the same time, the flocs could adsorb microalgae cells, prevent them from losing in the reaction process, thereby maintaining the structural stability of the microalgae–bacteria consortium [78]. *Chlorella* was cultured in unsterilized seafood wastewater, and the flocculation activity was 92.0% ± 6.0%, which was much higher than that of sterilized seafood wastewater (8.7% ± 2.5%) [104].

### Application of microalgal biomass

Through biochemical or thermochemical conversion, microalgal biomass can be applied in biofuel, feed, food, fertilizer, biochar and pharmaccutical biocompound.

#### Biofuel production

Algal biomass is capable to produce biofuels, including biodiesel, biogas, bioalcohols, biohydrogen, biosynfuel, and bio-oils [114, 115]. For example, the lipid content produced by cyanobacteria reached 12.74%, and the obvious dominance of C14 and C18 fatty acids in the total lipid content indicates their applicability as potential biofuels [23]. The residual microalgal biomass after lipid extraction were further processed by anaerobic digestion to produce biogas [116]. *Chlamydomonas* sp. QWY37 contained high content of carbohydrate in their cells, and the carbohydrate can be transformed to ethanol by applying engineered yeast [117]. Through anaerobic solid-state fermentation and the subsequent light independent fermentation, microalgal biomass was transformed to biohydrogen [118]. Lunprom et al. is reported that this sequential process produced 16.2 mL⋅H_2_/gvs [118].

#### Feed and food

Microalgae are rich in nutrients (e.g., vitamins, polysaccharides, mono- and polyunsaturated fatty acids, minerals, etc.) [67]. Therefore, cultured microalgae have been widely used in animal feed [119]. Qureshi et al. incorporated *Spirulina platensis* into poultry feed and improved the yellowness of the skin and yolk of broilers [120]. Thaakur et al. found that adding *Spirulina plantensis* to feed can help improve the antioxidant level of animal tissues [121].

Moreover, microalgae have been directly used in complementary food for humans (such as baked food, snacks, beverages, yogurt etc.), and its extracts can be produced as tablets or capsules as functional foods [99]. For example, *Spirulina plantensis* has much substances with biological activities to achieve antioxidant, antiviral, antibacterial, immune regulation, and cancer suppression. *Rhodococcus pluvialis* is rich in natural astaxanthin, which has multiple effects, such as anti-aging, relieving fatigue, and preventing cardiovascular and cerebrovascular diseases. Fernando et al. documented that ~ 49.3% TN, ~ 50.9% COD, and ~ 69.4% TP were reduced by *Haematococcus pluvialis* in industrialized run-off, and ~ 22.43 mg/L of astaxanthin were produced from these *Haematococcus pluvialis* [122]. It should be noted that microalgae grown in wastewater or waste gas may absorb some pollutants in cells, thereby affecting their usage as feed or food.

#### Fertilizer

The application of microalgal fertilizer is able to (1) improve the physical and chemical properties of soil, and (2) enhance the quality and yield of crops grown [123, 124]. However, the application of microalgae fertilizers is in the laboratory research stage. Sharma et al. reported that the addition of microbial fertilizers (algae biofilm and algae) increased the chlorophyll concentrations of soil, enhanced the content of polysaccharide and protein in corn as well as the length of cob [123]. Through field experiments, Dineshkumar et al. found that the content of pigment, total soluble sugar, and total free amino acid in onion grown in treatment with the addition of microalgae and cow manure are higher than those in onion grown in control group with only cow manure [124]. In wastewater, the biomass of mixed microalgae (*Chlorella vulgaris* and *Scenedesmus* sp.) reached 1.78 g·L^−1^ [125]. The combination of their residue after extracting oil and inorganic fertilizers in a ratio of 1:1 increased the yield of *Solanum lycopersicum* by 1.74 times [125].

#### Biochar

The applications of microalgal biochar have been explored from the following aspects: (1) improve soil fertility for agricultural purpose, (2) remediate wastewater or soil, (3) develop carbon electrode catalyst, and (4) manufacture energy storage [126, 127]. For example, *Enteromorpha prolifera* biochar was used to repair coastal saline–alkali soil in Wu et al.’s study, the best soil improvement effect was achieved when the addition amount was 1.5% and pyrolysis temperature was 400 ℃ [127]. Khan et al. reported that the selective modification of microalgae biochar can remove the targeted removal of contaminants effectively [128]. Compared to graphite plate electrodes, algal bloom-derived biochar used as an anode has high adsorption and stronger electrochemical response to redox media [129]. Compared with traditional heat treatment to obtain algal biochar, the treatment duration (20 min) of microwave mediated low-temperature treatment is shortened with obtaining 73.3% carbon [130].

#### Pharmaceutical bio-compounds

Owing to the bioactive nature of carbohydrates in algal cells, many algal strains are applied widely in the pharmaceutic industries, such as *Chlorella*, *Spirulina*, *Griffithsia*, and Diatoms etc. [131, 132]. High-value compounds from these microalgae inhibit antimicrobial, antifungal, anti-cancer, and antiviral activities [132]. For example, several antiviral agents have been extracted from the microalgal biomass. A protein cyanovirin–N derived from *Nostoc elipsosporum*, a sulfated polysaccharide calcium spirulan obtained from *Spirulina platensis*, *Gigartina skottsbergii* synthesized from marine algae, and carrageenan and chitosan polysaccharides from algae have been applied in inhibiting the replication of a wide variety of viruses [114, 133–135].

## Challenges and prospects

**Fig. 7:**
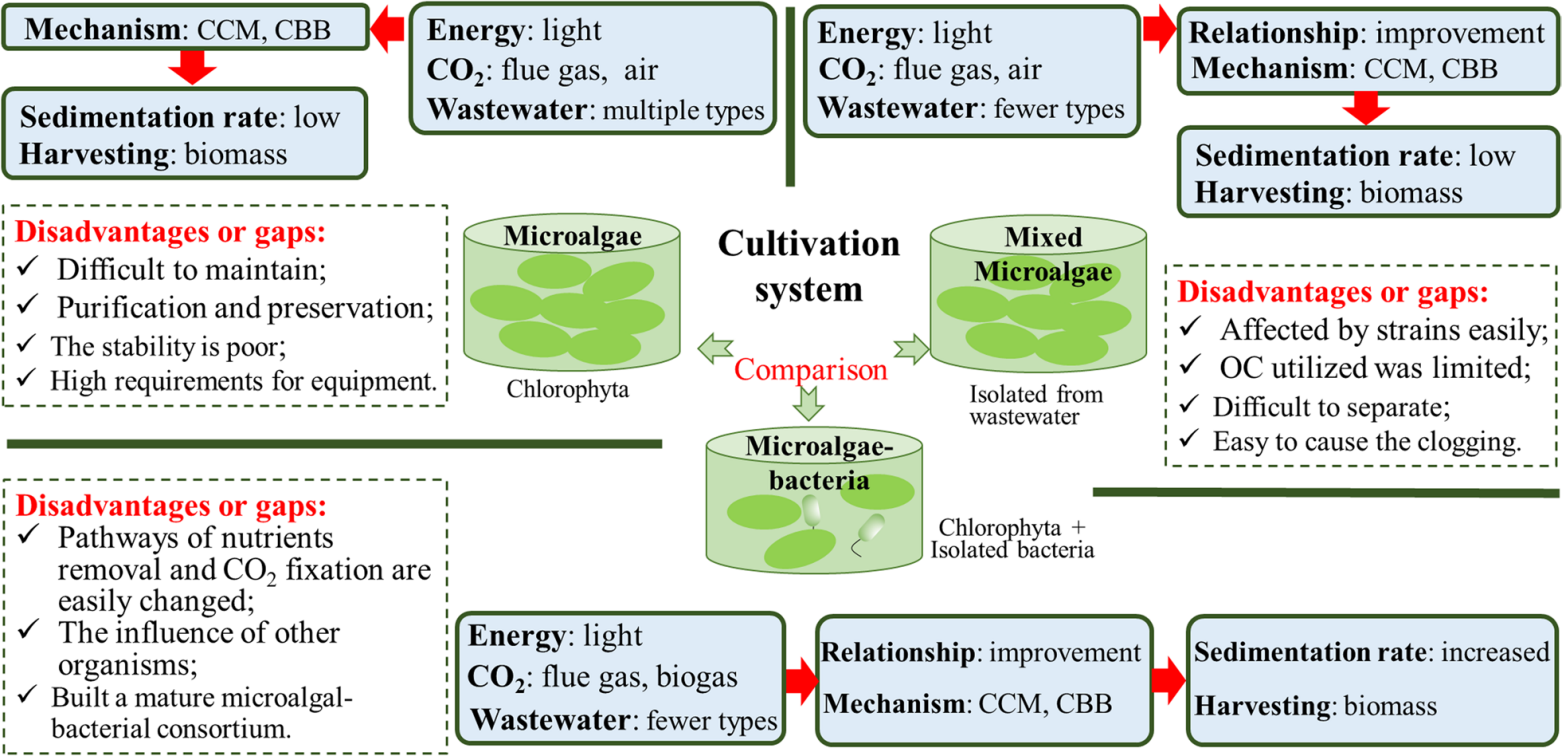
Comparison of application of microalgae and microalgae–bacteria consortia in fixing CO_2_ coupled with wastewater purification

The application of single microalgae, mixed microalgae and microalgae–bacteria consortia in fixing CO_2_ coupled with wastewater purification was discussed and compared in detail, as shown in Fig. 7. Different methods should be selected according to the specific goals, cultivation conditions, advantages and disadvantages. The challenges and prospects in the applications and commercialization of these microorganisms are summarized below.

(1) As carbon and nutrients source, the composition of flue gas and wastewater produced at different conditions are different. The reliance of microalgae on the varied composition of flue gas and wastewater was the main challenge hindering their application for microalgae cultivation. To address this challenge, microalgae with strong adaptability to environment and high CO_2_ fixation ability can be selected. In addition, bacteria that could promote the growth of beneficial microalgae can be screened.

The current genomic, transcriptomic, proteomic and metabolomic knowledge of microalgae would provide key information for the improvement of the biomass production and biotechnology processes. The regulation of the interaction of microalgae and bacteria in the consortia should be investigated at the molecular level to establish synergy among the cultured microorganisms and improve the overall efficiency of CO_2_ fixation and wastewater treatment.

(2) Because most of these studies were performed in laboratory units, they were not applied in scaled-up conditions with different system capacities and external factors. The application of AI technologies in adjusting microalgal CO_2_ fixation system is still in initial stage. Future studies will require large-scale outdoor experiments with AI technologies to assess the economic viability and sustainability of these biotechnological applications.

(3) Microalgae CO_2_ fixation technology is still limited by the high costs of system construction, CO_2_ gas transportation, microalgae cultivation and biomass harvesting. Therefore, it is important to develop cost-effective and efficient extraction and harvesting technologies. Meanwhile, the export of microalgae products is mainly based on microalgae powder, while the proportion of deeply processed microalgae products is relatively small. Thus, researchers also could delve into potential uses of microalgal biomass and further shorten the processing stage of microalgae in various applications to generate income from microalgae for long-term sustainability and environmental benefits.

## Conclusions

Microalgae and microalgae–bacteria consortia have broad prospects in CO_2_ fixation, nutrient removal, and resource utilization. The current goal is to reduce the gaps between the expanding microalgae studies and the related applications by exploring relevant mechanisms, screening and testing adaptable microalgae and bacteria, adjusting suitable cultivation conditions, and obtaining sufficient meaningful data. The reported work and the emergent challenges in the application of single microalgae, mixed microalgae, and microalgae–bacteria consortia were reviewed. Because specific microalgae strains contain high-value products that are desired for harvest, most cultures of algae are currently grown as monocultures. In contrast, a mixed microalgae and microalgae–bacteria consortium may mitigate environmental risk, obtain high biomass, and improve the efficiency of nutrient removal. The mechanisms of nutrient removal and CO_2_ fixation by microalgae and microalgae–bacteria consortia were also emphasized, and the importance of microalgae was proven. However, the application of microalgal biomass is still in the exploratory stage. Although there are numerous benefits in cultivation of microalgae–bacteria consortium by waste gas–waste water, their industrialization and commercialization still face some challenging obstacles. This paper provided guidance on future work to support the development of CO_2_ fixation coupled with nutrient removal by microalgae and microalgae–bacteria consortia.

## Data Availability

The data that support the findings of this study are available on request from the corresponding author.
